# First responder systems can stay operational under pandemic conditions: results of a European survey during the COVID-19 pandemic

**DOI:** 10.1186/s13049-022-00998-3

**Published:** 2022-02-19

**Authors:** Camilla Metelmann, Bibiana Metelmann, Michael P. Müller, Bernd W. Böttiger, Georg Trummer, Karl Christian Thies

**Affiliations:** 1 Department of Anaesthesiology, University Medicine Greifswald, Ferdinand-Sauerbruch Straße 1, 17475 Greifswald, Germany; 2 Department of Anaesthesiology, Intensive Care and Emergency Medicine, St. Josefskrankenhaus, Sautierstraße 1, 79104 Freiburg im Breisgau, Germany; 3 Department of Anaesthesiology and Intensive Care Medicine, Faculty of Medicine and University Hospital Cologne, Kerpener Straße 62, 50937 Köln, Germany; 4 Department of Cardiac and Vascular Surgery, University Cardiac Centre Freiburg – Bad Krozingen, Hugstetter Straße 55, 79106 Freiburg, Germany; 5 Klinik für Anästhesiologie, EvKB, Universitätsklinikum OWL der Universität Bielefeld, Campus Bielefeld-Bethel, Burgsteig 13, 33617 Bielefeld, Germany

**Keywords:** Resuscitation, Cardiac arrest, OHCA, First responder, Community first responder, Citizen first responder, Covid-19, Pandemic, Personal protective equipment, Survey

## Abstract

**Background:**

Dispatching first responders (FR) to out-of-hospital cardiac arrest in addition to the emergency medical service has shown to increase survival. The promising development of FR systems over the past years has been challenged by the outbreak of COVID-19. Whilst increased numbers and worse outcomes of cardiac arrests during the pandemic suggest a need for expansion of FR schemes, appropriate risk management is required to protect first responders and patients from contracting COVID-19. This study investigated how European FR schemes were affected by the pandemic and what measures were taken to protect patients and responders from COVID-19.

**Methods:**

To identify FR schemes in Europe we conducted a literature search and a web search. The schemes were contacted and invited to answer an online questionnaire during the second wave of the pandemic (December 2020/ January 2021) in Europe.

**Results:**

We have identified 135 FR schemes in 28 countries and included responses from 47 FR schemes in 16 countries. 25 schemes reported deactivation due to COVID-19 at some point, whilst 22 schemes continued to operate throughout the pandemic. 39 schemes communicated a pandemic-specific algorithm to their first responders. Before the COVID-19 outbreak 20 FR systems did not provide any personal protective equipment (PPE). After the outbreak 19 schemes still did not provide any PPE. The majority of schemes experienced falling numbers of accepted call outs and decreasing registrations of new volunteers. Six schemes reported of FR having contracted COVID-19 on a mission.

**Conclusions:**

European FR schemes were considerably affected by the pandemic and exhibited a range of responses to protect patients and responders. Overall, FR schemes saw a decrease in activity, which was in stark contrast to the high demand caused by the increased incidence and mortality of OHCA during the pandemic. Given the important role FR play in the chain of survival, a balanced approach upholding the safety of patients and responders should be sought to keep FR schemes operational.

**Supplementary Information:**

The online version contains supplementary material available at 10.1186/s13049-022-00998-3.

## Background

In cardiac arrest cardiopulmonary resuscitation (CPR) needs to start as soon as possible to achieve survival with good neurological outcome [1]. The first minutes of cardiac arrest are of vital importance [2] and survival can be doubled to tripled, if CPR starts before arrival of the emergency medical service (EMS) [3, 4]. During the last decade many different systems were implemented to dispatch first responders (FR) to out-of-hospital cardiac arrests (OHCA) [5–12]. FR are usually alerted by smartphone and arrive earlier on scene than the EMS, leading to an earlier start of CPR [6, 13–15]. Approximately half of all European countries have implemented—to some degree—FR schemes [16] and the number is growing fast. FR schemes are associated with increased survival [7, 17–19]. A European registry study indicates, that regions operating such systems have significantly higher OHCA survival rates than other European regions [16]. The American Heart Association guidelines 2020 as well as the European Resuscitation Council (ERC) Guidelines 2021 strongly encourage the implementation of FR systems [20, 21].

This promising development of FR systems has been challenged by the outbreak of Coronavirus Disease 19 (COVID-19) [22, 23]. The pandemic has affected all links of the chain of survival, which called for operational adaptations to protect patients and responders [24–27].

During the pandemic a significant increase of OHCA rates were noted [28]. The incidence correlated closely with peaks of the COVID-19 incidence [29]. At the same time, bystander initiated CPR decreased significantly [24, 30].

During the pandemic survival and favourable neurological outcome after OHCA have decreased [31, 32], which would ask for an expansion of first responder programmes. At the same time however, safety of first responders and the risk of contracting COVID-19 on a mission must be considered [22, 26, 33, 34]. Hence, the benefits of dispatching first responders have to be balanced against the risk of exposing first responders and patients to COVID-19 [22, 34, 35].

The primary goal of this study was to understand the impact of the pandemic on First Responder schemes throughout Europe. The secondary goal was to scope the measures taken by the operators to minimise the infection risk for patients and responders.

## Methods

A questionnaire-based descriptive cross-sectional study was done by members of the ERC Research NET during the second wave of the COVID-19 pandemic in Europe.

### Questionnaire

The questionnaire (Additional file 1) was developed by the three authors MPM, BM and CM. A content validity analysis of the questionnaire was performed by the authors KCT, BB and GT. The questionnaire was circulated in English and consisted of 37 single-choice, multiple-choice, as well as open-phrased questions. The questionnaire captured general characteristics of the participating FR schemes, information on the impact of the pandemic on the schemes and their corresponding reactions to COVID-19.

### Participants

Our search strategy included all 53 European countries, as defined by the World Health Organization. FR schemes were identified in a two-step approach: First by a literature search for publications on European FR schemes, that dispatch first responders to OHCA. Metadata about the publications and the corresponding authors were collected. Second, an extensive internet search for FR systems was performed for each European country.

Our literature search revealed 49 researchers from 18 countries. The internet search identified 135 responder systems in 28 countries (53% of all European countries). Additional file 2 presents a break-down of the results. A total of 206 invitations were sent. If systems provided more than one person as contact on their web pages, all were contacted. Hence, the number of invitations sent exceeded the sum of identified researchers and FR systems.

### Exclusion criteria

Responses from FR systems that were not fully implemented at the time of the survey were excluded. Responses from outside Europe were excluded. Incomplete replies were also excluded if key questions regarding COVID-19-management were not answered. We have only included one response per scheme. If more than one response per scheme was received, we checked the questionnaires for consistency and omitted diverging answers.

### Distribution of questionnaire

The online platform SurveyMonkey was used. Personalised invitations were sent via email. The study was conducted over a two-month period from December 11th 2020 to February 11th 2021. To increase the response rate, a reminder was sent on January 16th 2021 to all schemes, that had not answered (n = 84).

### Data analysis

Descriptive statistics (absolute and relative frequency) were used to evaluate individual questionnaire items. Answers given to open questions were either directly quoted or processed using quantitative content analysis.

## Results

We received 55 responses from 135 FR systems in 18 countries, resulting in a response rate of 41%. Eight responses were excluded from further analyses: one scheme was not located within Europe; one region had not fully implemented a FR system at the time of the survey; four participants did not answer key questions of the questionnaire; and for two schemes more than one response was received.

Responses from 47 regions in 16 European countries (35% of all contacted FR systems) were included in the analysis (Table 1).

**Table 1:** Countries and regions, from which first responder systems responded to the survey

Country	Region	Number of km^2^ covered	Number of inhabitants covered	Number of first responders
Albania	Tirana	502	800,000	68
Austria	Vienna	415	1,900,000	n.a
Belgium	Hoogstraten	105	21,000	250
Denmark	Nationwide	42,944	5,800,000	103,658
	Southern Denmark	12,191	1,223,000	500
	Faroe Islands	1190	53,000	289
Finland	Pirkanmaa	14,000	515,000	50 units*
France	Moselle	6216	1,000,000	2100
Germany	Aachen	160	250,000	1255
	Berlin	792	3,700,000	4000
	Bielefeld	258	330,000	600
	Duisburg	233	495,000	260
	Emsland	3700	481,000	1900
	Freiburg/ Breisgau	1531	493,000	1010
	Groß-Gerau (county)	453	270,000	300
	Gütersloh (county)	968	366,000	750
	Hochsauerlandkreis	1960	259,777	743
	Peine (county)	535	135,000	200
	Osnabrück (city and county)	2200	520,000	1400
	Stormarn (county)	766	250,000	2 units
	Vorpommern-Greifswald	3927	235,623	411
Ireland	Nationwide	70,000	4,500,000	200
	Wicklow	202	5000	16
Italy	Emilia Romagna	22,500	4,400,000	9900
Luxembourg	Nationwide	2500	630,000	56 units
	Luxembourg (city)	50	15,000	20
Netherlands	Nationwide	41,543	17,440,679	300,000
Romania	Bucharest-Ilfov	2000	5,000,000	3000
Slovenia	Nationwide	5000	600,000	3300
Sweden	Region Blekinge	3039	159,684	2965
	Region Kronoberg	8466	199,886	2141
	Region Östergötland	10,562	467,095	4118
	Region Sörmland	6060	299,101	3712
	Region Stockholm	6519	2,389,923	24,487
	Region Västmanland	5146	277,074	3118
	Region Västra Götaland	23,942	1,733,574	19,167
Switzerland	Basel-city and Baselland	550	500,000	1417
	Bern	5960	1,000,000	2400
	Geneva	16	500,000	975
	Kanton St. Gallen	2031	510,000	500
	Obwalden	400	30,000	150
	Ticino Region	2812	360,000	4500
	Wallis	5200	350,000	1200
United Kingdom of Great Britain and Northern Ireland	Bassetlaw	638	120,000	30 units
	Northamptonshire	16,000	500,000	35 units
	Surrey	225	323,960	8 units
	Woking, Surrey	20	100,000	10 units

n.a. indicates “not answered”; * some regions stated number of “first responder units” instead of number of participants. “Units” often represent one car/shift; all numbers were provided by survey participants

As illustrated in Table 2, FR schemes are organised differently throughout Europe: We found heterogenous alerting modes, varying minimum qualifications of first responders as well as differing maximum numbers of first responders dispatched per mission. There also was no uniform approach regarding the use of automated external defibrillators (AED).

**Table 2:** Characteristics of first responder systems

Question	Answer	n	%
How do you alert the first responders?	Telephone alerting system	2	4
	Pager	1	2
	SMS alerting system	5	11
	Smartphone based alerting system	31	66
	Combination of SMS and smartphone based alert	7	15
	Other^§^	1	2
What's the minimum qualification of your first responders?	No specific qualification	3	6
	BLS course or first aid course or equivalent	38	81
	Higher than BLS (e.g. nurse, medical doctor, paramedic)	6	13
What is the maximum number of responders you dispatch per mission?*	1	3	7
	2–3	20	45
	4–5	6	14
	6–10	0	0
	11–20	3	7
	> 20	11	25
	I don´t know	1	2
Do you dispatch responders to fetch AED?*	Yes	26	62
	No	16	38

^*^Due to missing answers n does not always add up to 47; ^§^ Pager plus SMS

### Impact of the pandemic on FR systems

**Fig. 1:**
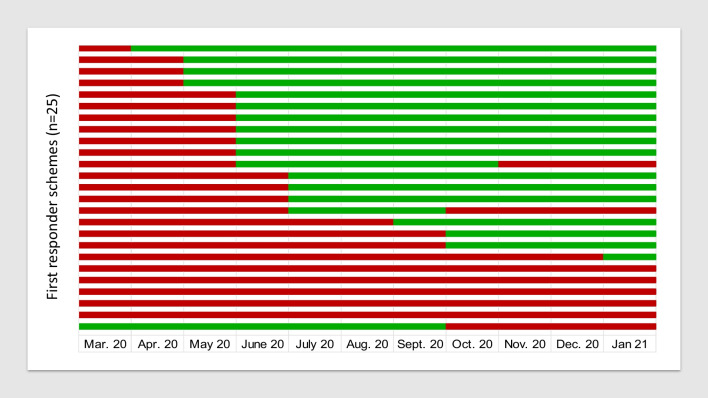
Months in which the 25 FR schemes, which stopped due to COVID-19, did not operate (marked in red) and operated (marked in green)

Out of the 47 schemes, 25 (53%) reported, that they had deactivated their system due to COVID-19 at some point but 22 (47%) continued without a break. While most systems stopped in March 2020 at the beginning of the first wave, one system continued but stopped in October 2020 with rise of the second wave. Two systems stopped again at the beginning of the second wave (Fig. 1). The duration of deactivation differs: some stopped for one or two months, others continued to be suspended at the time of the survey (December 2020 till February 2021). Reasons for stopping the system were described as lack of knowledge about the new virus, the perceived high risk of transmission as well as lack of personal protective equipment (PPE). While 21 FR schemes reported falling numbers of accepted call outs compared to the 3 months prior to the pandemic, 10 did not see a change and 2 reported an increase. 10 FR schemes could not say whether there was a change in their response rate. Similarly, 18 FR schemes saw fewer registrations of new volunteers since beginning of the pandemic. 13 reported no change and 5 reported an increase of applications. 8 did not know. Some schemes reported, that despite provision of adequate PPE a large percentage of FR stopped responding to alerts.

Out of the 47 responding schemes, six (13%) were aware of responders, that contracted COVID-19 on a mission. Two (4%) systems were aware of cases within their scheme, in which first responders transmitted COVID-19 during a mission. Six regions (13%) reported, that their COVID-19 management was criticised.

### Protective measures during the pandemic

Respondents reported a mixed incidence of COVID-19 cases per 100,000 inhabitants within the week preceding the survey, ranging from 3 to 1500 cases (median 209).

While 36 systems (77%) reported a uniform approach within their scheme, one system reported different incident based regional approaches during the COVID-19-pandemic.

In the wake of the pandemic 31 FR schemes (66%) changed their general management to ensure the safety of their responders. Reported measures were: providing health and safety information on COVID-19; issuing PPE; switching to hands-only CPR; not dispatching to patients with suspected/confirmed COVID-19; limiting the range of indication for FR; reducing the number of responders dispatched per mission, or excluding first responders at risk (e.g. age or diseases), and shortening the time at scene. To achieve this, a closer collaboration with the EMS was sought. FR were encouraged to perform an individual risk assessment and to reject alarms, if they felt sick or had any concerns regarding their own health. Many FR schemes put a stronger focus on information of their responders and reported expanding eLearning or sending non-critical notifications offering advice/information on COVID-19 through their app systems. Some schemes have intensified fund raising to allow for provision of PPE.

To reduce rescuers’ exposure risk, eight systems (17%) have limited the number of first responders dispatched to an OHCA.

**Fig. 2:**
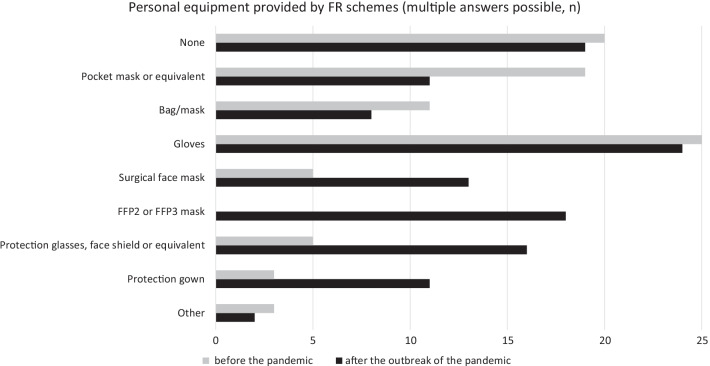
Personal equipment provided by FR schemes before and after outbreak of COVID-19 (n)

The personal equipment issued to FRs differed (Fig. 2). Gloves were supplied by 25 (53%) systems before and 24 (51%) after COVID-19 outbreak. Increased provision of surgical face masks, protection glasses, face shields or equivalent, and protection gowns was reported. FFP2 or FFP3 masks (filtering face piece; high performance face masks complying with European Standard EuroNorm 149) were introduced after the COVID-19 outbreak in 18 (38%) schemes. Further equipment issued were plastic aprons, vests, laryngeal tubes and also hand disinfectant. No personal equipment was provided by 20 systems (43%) before COVID-19 outbreak and 19 systems (40%) after the outbreak.

Twenty-four (51%) schemes reported, that their responders had received instructions on the use of personal protective equipment (PPE). Thirty-nine (83%) systems communicated a pandemic-specific algorithm to their responders. Thirty-eight (81%) of these algorithms were based on ERC COVID-19 guidelines [34] or other official guidelines.

Table 3 illustrates the different approaches during the pandemic in relation to a scheme's percentage of first responders employed in health care. Systems with a lower percentage of FR employed in health care had a higher tendency to recommend hands-only-CPR prior to COVID-19, teach FR how to use PPE and communicate a pandemic-specific algorithm.

**Table 3:** Strategies chosen in relation to regions´ percentage of first responders employed in health care

		How many of your responders are employed in health care?			
		< 25%	25–50%	50–75%	> 75%
Number of FR regions		17	9	12	4
Did you recommend hands-only-CPR to your first responders prior to COVID-19?*	Yes	9	4	3	1
	Yes, but only to untrained first responders	1	2	1	1
	No	5	3	8	2
Did you teach your first responders how to use personal protective equipment?	Yes, we sent them information material/links/videos	10	3	2	1
	Yes, we have provided hands-on training	5	1	0	0
	No	2	5	10	3
Was the system deactivated due to COVID-19 at any point?	Yes	10	4	8	2
	No	7	5	4	2
Was a pandemic-specific algorithm communicated to the first responders?*	Yes	14	9	12	1
	No	1	0	0	2

5 FR regions did not indicate their percentage of FR employed in health care. *differences to n = 42 are caused by missing answers

## Discussion

Dispatching first responders to OHCA strengthens the chain of survival and increases the likelihood of good neurological outcome [18, 19, 36]. This study illustrates the impact of the pandemic on FR schemes and gives an overview over the corresponding measures taken to mitigate the risks for responders and patients. There is a remarkable diversity between first responder schemes in Europe [5, 10–12, 37]: different minimum qualification of responders, different percentages of professional health care providers within the schemes, different modes of activation, different maximum numbers of FR dispatched per mission and different approaches to AED use. It is therefore not surprising that first responder systems took different approaches, when faced with COVID-19 [22, 38]. One system even took different regional measures (based on COVID-19 incidence) within their scheme. To reduce risk of transmission, three major strategies were identified: (i) suspension of the system, (ii) reduction of number of exposed responders (by reducing the number of FR dispatched and/or limiting the indications for deployment to OHCA and unconsciousness) and (iii) provision of PPE. A combination of these strategies was often chosen. Several systems paused operations for some time to distribute PPE. Timepoints chosen for reopening differed. PPE issued varied between schemes and only some systems provided information or training on the use of PPE. Systems working with both professional first responders (fire fighters and police) as well as lay citizen often differentiated their management between these groups. Most systems communicated pandemic-specific algorithms to their responders. These algorithms were nearly always based on the ERC COVID-19 guideline [34] or national guidelines.

The need to teach FR how to use PPE and to communicate a pandemic-specific algorithm appeared to be less, when the majority of FR were health professionals. However, in a survey conducted among Italian physicians, nearly half of them stated, that the information on the use of PPE they had received, was not sufficient [39]. Half of the schemes with more than 75% of FR being health care professionals, deactivated the systems during the pandemic. This could have been done to protect this scarce workforce from contracting COVID-19, or because their presence was urgently needed at their primary workplace.

The pandemic had a negative impact on numbers of accepted call outs and new FR registrations [40]. The willingness of laypersons to perform CPR decreased during the pandemic, whereas provision of PPE counterbalanced this effect [41].

The dynamic situation and wide variety in local COVID-19 incidences (3–1500 cases per 100,000 inhabitants within the week preceding the survey) requires protocols to be tailored to the local conditions. Protocols and procedures should be evaluated and adapted regularly in line with the regional epidemiology and evolving scientific evidence [22, 34, 42, 43]. In the prehospital environment COVID-19-status is mostly unknown [26]. COVID-19 infections are not easy to detect, because initial symptoms are unspecific and only occur after an incubation period; in young and healthy persons symptoms can even be inapparent [43–45]. Despite all precautionary measures, the risk of contracting COVID-19 during CPR cannot be eliminated [46]. Several systems reported that they are aware of first responders, who contracted or transmitted COVID-19 during a mission. Therefore, responders should be fully vaccinated and provided with PPE [34, 47, 48].

The results of our study demonstrate a huge variation in impact of the pandemic on FR schemes and correspondingly a huge variation in precautionary measures for providers and patients; however, these variations are not only caused by the heterogeneity of the responder systems, but also by a comprehensible lack of knowledge around the new viral disease, lack of experience in dealing with highly contagious infectious diseases, and a lack of guidance on how to deal with this unprecedented challenge [49]. This led the majority of systems to suspend their operations at some point. However, the soaring rate of OHCA, the plummeting outcomes as well as the decreased rate of bystander CPR during the pandemic require the FR systems to remain operational [38]. The examples of 'good practice' we have found amongst the respondents demonstrate that the infection risk can be managed and that the challenges the pandemic poses to FR schemes could be overcome [35]. The majority of FR schemes reported that they experienced no criticism regarding their management during COVID-19.

With the current knowledge of COVID-19 we recommend that only fully vaccinated responders should be dispatched, that they should be issued with PPE, particularly FFP2 masks, face shields or goggles and gloves. Special resuscitation training focusing on the particularities of the modified COVID-19 CPR algorithm should be made available for all responders. PCR testing of all cardiac arrest victims treated by FR should be mandatory. There also should be a continuous regional risk assessment for FR schemes taking local spread and virus variants into consideration.

### Limitations

Due to the dynamic nature of the pandemic our research was done in English, which has most likely introduced a language bias. We assume that many first responder systems may not have published in English nor have a web presence in English and therefore remained undetected by our search.

Additionally, our invitations as well as the survey itself were in English. This could have led to reduced participation and underrepresentation of non-english speakers. Nevertheless, most helpful responses could be collected from 18 countries. A response rate of 41% is common for an online survey among health care professionals [50]. Due to search strategy used, organisations or mail addresses might have been identified, which were no longer in operation.

When scrutinising the results, it has to be kept in mind, that each FR scheme is weighted equally and that we made no adjustments for the numbers of first responders or inhabitants covered by each system. Additionally, out of the 47 included FR schemes thirteen were located in Germany, seven in Sweden and seven in Switzerland. These three countries represent 57% of all responses. While this overrepresentation might decrease the external validity, it reflects the current situation, that in most European countries several FR schemes co-exist and are organised and managed independently from each other. Since FR systems reacted differently to COVID-19 even within one country, the data are presented separately for each FR system and not on a national level.

## Conclusions

First responder systems are organised and operate in different ways throughout Europe. The unprecedented spread of the pandemic has led the majority of FR schemes to pause operations to protect responders and patients. The ever-evolving knowledge about COVID-19 has not allowed to develop a universal strategy for first responder schemes. Nevertheless, we have identified approaches that could serve as templates of good practice and help to keep FR schemes operational throughout the pandemic.

## Supplementary Information

**Additional file 1**: Questionnaire

**Additional file 2**: European countries with identified first responder systems

## Data Availability

Data generated and analysed during this study are included in this published article. Raw data are available from the corresponding author on reasonable request.

## Abbreviations

CPR: Cardiopulmonary resuscitation
COVID-19: Coronavirus Disease 19
EMS: Emergency medical service
ERC: European Resuscitation Council
FFP 2 or FFP 3 mask: Filtering face piece mask category 2 or 3
FR: First responders
OHCA: Out-of-hospital cardiac arrest
PPE: Personal protective equipment
