# Supplementary material for: First responder systems can stay operational under pandemic conditions: results of a European survey during the COVID-19 pandemic
Source: Scand J Trauma Resusc Emerg Med. 2022 Feb 19;30:10. doi: 10.1186/s13049-022-00998-3 (PMC8857892; doi:10.1186/s13049-022-00998-3)
Supplement: Supplementary file 1 — Additional file 1: Questionnaire [file 13049_2022_998_MOESM1_ESM.pdf]

Impact of the Covid-19 pandemic on Community First Responder Systems

We very much appreciate your effort and your time to answer this survey.

Your participation in this research study is voluntary. You may withdraw at any time or decide not to answer any specific question. Your responses will be confidential. The survey was approved by the ethical committee Greifswald University Medicine, Germany. By completing this survey, you are consenting to participate in this study.

NB: Some first responder schemes cover large areas with uneven regional population densitities and variations in infrastructure. This may require different operational responses towards Covid-19 within the same scheme. ***If you are operating different regional policies within your scheme we would appreciate if you could fill in the survey for each region separately and specify the region, your answers apply to.***

* 1. In which country is your first responder system located?

2. In which region (city/county/district) is your system based?

3. How many citizens live in your area and are covered by your scheme?

4. How many km^2 does your region cover approximately?

5. How many first responders take part in your scheme?

6. How many new Covid-19 infections in your region per 100.000 inhabitants were counted in the last 7 days?

7. Have you differenciated between regions within your scheme during Covid-19-pandemic e.g. based on numbers of infected persons?

- ◯ Yes
- ◯ No

8. How do you alert the first responders?

- ◯ Telephone alerting system
- ◯ Pager
- ◯ SMS alerting system
- ◯ Other
- ◯ Smartphone based alerting system
- ◯ Combination of SMS and smartphone based alert

9. Which software/ app do you use?

10. What's the minimum qualification of your first responders?

- ◯ No specific qualification
- ◯ BLS course or first aid course or equivalent
- ◯ Higher than BLS (e.g. nurse, medical doctor, paramedic)

11. How many of your responders are employed in health care?

- ◯ Less than 25%
- ◯ 25-50%
- ◯ 50-75%
- ◯ More than 75%
- ◯ I don't know.

12. What is the maximum number of responders you dispatch per mission?

13. Did you recommend hands-only-CPR to your first responders prior to Covid-19?

- ◯ Yes
- ◯ Yes, but only to untrained first responders
- ◯ No

14. What personal equipment did you provide for your first responders **before the pandemic**? (multiple answers possible)

- ☐ None
- ☐ Pocket mask or equivalent
- ☐ Bag/mask
- ☐ Gloves
- ☐ Surgical face mask
- ☐ FFP2 or FFP3 mask
- ☐ Protection glasses, face shield or equivalent
- ☐ Protection gown
- ☐ Other

15. What personal equipment did you provide for your first responders **after the outbreak of the pandemic**? (multiple answers possible)

- ☐ None
- ☐ Pocket mask or equivalent
- ☐ Bag/mask
- ☐ Gloves
- ☐ Surgical face mask
- ☐ FFP2 or FFP3 mask
- ☐ Protection glasses, face shield or equivalent
- ☐ Protection gown
- ☐ Other

16. Did you teach your first responders how to use personal protective equipment (PPE)?

- ◯ Yes, we sent them information material/links/videos
- ◯ Yes, we have provided hands-on training
- ◯ No

* 17. Was the system deactivated due to COVID-19 **at any point**?

- ◯ Yes
- ◯ No

18. Why was the system deactivated?

19. When and for how long was the system deactivated?

20. Have you stopped again?

- ◯ No
- ◯ Yes, at (date)

21. Do you plan to restart?

- ◯ Yes
- ◯ No, because

22. When will you restart?

23. Was a pandemic-specific algorithm communicated to the first responders?

- Yes
- No

24. Is the pandemic-specific algorithm based on the ERC COVID-19 guidelines or any other official guideline?

- Yes
- No
- Other

25. How did the response rate of first responders change compared to the 3 months prior to the pandemic?

- Response rate decreased
- Unchanged
- Response rate increased
- I don't know.
- Other

26. How did the number of newly registered first responders per week/ month change since beginning of the pandemic?

- Decreased (you received less application per week/month)
- Unchanged
- Increased (you received more application per week/month)
- I don't know.

* 27. Are you aware of any first responders, who contracted Covid-19 during a mission?

- Yes
- No

* 28. Are you aware of any first responders, who transmitted Covid-19 during a mission?

- Yes
- No

29. Was your Covid19 management criticised?

- Yes
- No

30. Did Covid-19 change your general management of your first responder system?

- Yes, but very little
- Yes, immensely
- No

31. How did you change your general management?

32. Do you dispatch responders to fetch AED?

- Yes
- No

33. Did your first responders exceperience any difficulties in accessing AEDs during the lockdown?

- ◯ Yes
- ◯ No
- ◯ I don't know.
- ◯ We supply every first responder with an AED.

Management due to Covid-19

34. Did you limit the number of first responders dispatched to an OHCA to reduce rescuers’ potential viral exposure?

- ◯ Yes
- ◯ No

35. Have you taken any specific measures to protect your responders during the pandemic? (multiple answers possible)

- ☐ Yes, based on age
- ☐ Yes, based on previous deseases of first responder
- ☐ No
- ☐ Other

36. Do you dispatch first responder to suspected/known Covid-19-patients?

- ◯ Yes
- ◯ No
- ◯ I don't know.

37. Do you have any further comments?
