# Supplementary material for: First responder systems can stay operational under pandemic conditions: results of a European survey during the COVID-19 pandemic
Source: Scand J Trauma Resusc Emerg Med. 2022 Feb 19;30:10. doi: 10.1186/s13049-022-00998-3 (PMC8857892; doi:10.1186/s13049-022-00998-3)
Supplement: Supplementary file 2 — Additional file 2: European countries with identified first responder systems [file 13049_2022_998_MOESM2_ESM.pdf]

<u>European countries with identified first responder systems</u>

**Country**	**Number of contacted researchers***	**Number of contacted FR systems****
Albania	0	1
Andorra	0	1
Armenia	0	0
Austria	2	1
Azerbaijan	0	0
Belarus	0	0
Belgium	1	2
Bosnia and Herzegovina	0	0
Bulgaria	0	0
Croatia	0	1
Cyprus	0	0
Czechia	1	1
Denmark	4	2
Estonia	0	1
Finland	1	1
France	2	3
Georgia	0	0
Germany	3	37
Greece	0	0
Hungary	0	1
Iceland	0	0
Ireland	2	12
Israel	1	1
Italy	3	2
Kazakhstan	0	0
Kyrgyzstan	0	0
Latvia	0	0
Lithuania	0	0
Luxembourg	0	6
Malta	1	1
Monaco	0	0
Montenegro	0	0
Netherlands	3	1
North Macedonia	0	0
Norway	3	1
Poland	0	1
Portugal	0	1
Republic of Moldova	0	0
Romania	0	1
Russian Federation	0	1
San Marino	0	0
Serbia	0	0
Slovakia	0	0
Slovenia	1	1
Spain	1	1

Sweden	9	7
Switzerland	7	14
Tajikistan	0	0
Turkey	0	0
Turkmenistan	0	0
Ukraine	0	0
United Kingdom of Great Britain and Northern Ireland	4	32
Uzbekistan	0	0

Legend:

* who published on FR system in that country and were identified in literature search,

** whose contact data were identified in internet search
